# Robotic enterorrhaphy model for junior residents: A step closer to the fundamentals of robotic surgery manual skills assessment

**DOI:** 10.1007/s11701-026-03628-7

**Published:** 2026-07-20

**Authors:** Shayan Hosseinzadeh, Maria Carolina Jimenez, Michel Cordies, Tarini Mudunuri, Mahsa Shariat, Gary Girma Gamme, Jose Manuel Martinez, Robert Frank Cubas

**Affiliations:** University of Miami Hospital and Clinics, Miami, FL USA

**Keywords:** Robotic surgery, Surgical education, Simulation training, Enterotomy repair, Robotic suturing, Surgical skills acquisition

## Abstract

Robotic surgery is increasingly integrated into general surgical practice, creating a need for standardized training methods that objectively assess technical skill acquisition. Current robotic simulation curricula often rely on psychomotor tasks that may not reflect clinically relevant reconstructive procedures. Enterotomy repair requires precise tissue handling and suturing and may serve as a practical benchmark for robotic training. This study evaluated whether a standardized robotic enterotomy repair task could demonstrate reproducible skill acquisition among junior residents. A prospective longitudinal study was conducted involving PGY-1 and PGY-2 general surgery residents performing a standardized two-layer robotic enterotomy repair on a porcine small bowel model using the da Vinci Xi platform. Participants completed three sessions spaced 1–2 weeks apart. The primary outcome was time to task completion. Secondary outcomes included leak testing, technical errors, and exploratory comparisons by postgraduate year and prior robotic training. Changes in performance across sessions were analyzed using a linear mixed-effects model accounting for repeated measures. Thirty-eight residents participated, with 30 completing all three sessions. Mean completion time decreased from 18.47 ± 4.87 min in session 1 to 14.90 ± 6.00 min in session 2 and 13.20 ± 5.60 min in session 3 (*p* < 0.001). Leak rates declined from 26.3% in session 1 to 20.0% in session 3. Logistic regression analysis accounting for repeated observations demonstrated no significant association between completion time and leak occurrence (OR 1.0003, 95% CI 0.9988–1.0019; *p* = 0.671). PGY-1 residents demonstrated greater improvement, with convergence between PGY levels by the final session. Residents with prior robotic training demonstrated numerically faster repair times, though differences were not statistically significant. A short, standardized robotic suturing curriculum using a procedure-based enterotomy repair task produces reproducible improvements in technical efficiency among junior surgical residents without compromising repair integrity. This model provides an objective and practical framework for assessing robotic skill acquisition early in surgical training.

**Supplementary Information:**

The online version contains supplementary material available at 10.1007/s11701-026-03628-7.

## Introduction

Robotic surgery has become an integral component of modern general surgery, with expanding applications across colorectal, foregut, bariatric, and complex abdominal procedures [1, 2]. As robotic platforms proliferate, surgical trainees are increasingly expected to acquire robotic skills early in residency. However, formalized curricula for robotic skill acquisition remain heterogeneous, and objective, procedure-based assessments of competency are limited [3–5]. There is a paucity of standardized, reproducible methods to evaluate trainees’ ability to perform technically demanding robotic reconstructive tasks that mirror real operative challenges [4, 5].

Enterotomy repair represents a high-stakes skill that requires precise tissue handling, spatial awareness, and suture control, which are fundamental to performing safe robotic surgery [6]. Despite its clinical relevance, enterotomy repair has not been widely adopted as a benchmark task in robotic training curricula. Existing simulation assessments often emphasize basic psychomotor tasks or isolated suturing exercises, which may not adequately capture the complexity of layered bowel closure or the balance between efficiency and technical integrity [7–9].

Early exposure to structured robotic training has been proposed to accelerate skill acquisition and reduce variability in performance among trainees [10]. However, evidence supporting the effectiveness of short, focused robotic curricula, particularly those incorporating objective performance metrics and leak testing as a surrogate for repair quality, remains limited. Moreover, few studies have examined the reproducibility of learning curves across repeated sessions or explored whether gains in efficiency compromise technical outcomes [11].

To address these gaps, we designed a standardized robotic bowel suturing skills test incorporating a two-layer enterotomy repair with objective timing and leak assessment. This protocol was implemented as part of a short, structured robotic training curriculum for junior surgical residents. The primary aim of this study was to evaluate changes in time to completion of the task across repeated sessions as a measure of technical efficiency and learning. Secondary aims included assessment of repair integrity via leak testing, characterization of individual learning curves, and exploratory comparisons based on prior dedicated robotic training during residency and postgraduate training level.

We hypothesized that repeated participation in this standardized robotic suturing task would result in reproducible improvements in efficiency without compromising repair quality, and that junior trainees would demonstrate substantial gains over a short training interval. By providing a practical, procedure-based assessment framework, this study seeks to contribute objective evidence supporting structured robotic skills training early in surgical education.

## Methods and materials

### Study design and setting

This prospective, longitudinal study assessed performance of a standardized robotic bowel suturing task over three sessions, each spaced 1–2 weeks apart. All sessions were conducted on a Da Vinci Xi platform at the minimally invasive surgery skills lab at our institution. The principal investigator and co-investigator, both surgeons, oversaw the study, while a trained test administrator conducted the testing according to a standardized operating procedure.

This study was funded by a SAGES grant, and preliminary results were presented at the SAGES 2025 Annual Meeting. It was reviewed by the Institutional Review Board at our institution and was deemed exempt, as it involved an educational simulation study with trainee participants.

### Participants

Postgraduate year (PGY) 1 and 2 categorical and preliminary general surgery residents were eligible for participation. Residents were recruited on a voluntary basis. Participants completed up to three testing sessions, with residents who completed at least one session included in the analysis. Categorical PGY1 general surgery residents at our institution complete a dedicated month of training in the skills lab that includes robotic skills, while preliminary PGY1 residents receive similar training over a shorter, several-day period. Participant PGY level and prior participation in dedicated robotic training in the skills lab were recorded. Residents who had completed the dedicated one-month robotic skills laboratory rotation were classified as the trained group, while those who had not yet completed this rotation at the time of study participation, including all preliminary residents, were classified as the untrained group. This was an educational pilot study, and no formal sample size calculation was performed.

### Robotic setup and instrumentation

The robotic system was docked by the test administrator. The right robotic arm was equipped with a mega suture cut robotic needle driver, and the left arm with a standard robotic needle driver. The camera and instruments were checked for functionality before each session. Participants were seated at the robotic console and instructed to adjust ergonomic settings to their preference prior to beginning the task.

### Robotic bowel suturing task

Each testing session consisted of a standardized robotic enterotomy repair performed on a small intestine pig model. Prior to beginning the task, participants watched a standardized 7-minute instructional video outlining the procedural steps and expectations (Supplementary video).

A 10 cm segment of pig small bowel was prepared by the test administrator, and a 3 cm transverse enterotomy was created in the center of the segment. The bowel was positioned in the surgical field and secured at the four corners to prevent movement during suturing.

Participants performed a two-layer enterotomy repair in the following fashion:

First Layer: A full thickness running closure was performed in a baseball fashion using a 3 − 0 or 2 − 0 15 cm barbed suture. Bites were taken approximately every 5 mm and 5 mm from the cut edge, starting at the superior corner and proceeding to the inferior corner, terminating with a horizontal backward U stitch.

Second Layer: A second outer reinforcing layer was performed using three interrupted Lembert sutures placed at the two corners and the midpoint of the repair, approximately 1 cm from the cut edge. Each suture was secured with a surgeon’s knot followed by two square knots using 2 − 0 silk suture.

### Timing and task completion

Timing began when the participant grasped the needle with the robotic needle driver.

Upon completion of the first layer, the administrator immediately removed the barbed suture and introduced a 2 − 0 silk suture, 15 cm long, for the second layer. Timing was stopped after the participant completed the third knot of the final Lembert suture and cut the suture using the mega needle driver.

Total time to completion was recorded in minutes and seconds and used as the primary outcome measure.

### Leak testing

Following completion of the repair, the test administrator clamped both ends of the bowel segment using two bowel clamps. A 21-gauge needle connected to insufflation tubing was introduced through the bowel wall, and the bowel segment was submerged in water. Air insufflation was applied at 10 mm Hg, and the suture line was inspected for the presence of air bubbles for 10 s. This pressure was selected to approximate physiologic gastric endoluminal pressures during flexible upper gastrointestinal endoscopy (median ~ 10 mmHg), which are associated with adequate luminal distension and visualization [12].

Leak testing was recorded as a binary outcome (leak present vs. no leak) and served as a secondary measure of repair integrity.

### Repeated sessions

Each participant was invited to repeat the identical task at intervals of 1–2 weeks for a total of three sessions. All procedural steps, materials, instrumentation, and evaluation methods were kept constant across sessions to ensure standardization.

### Outcome measures

The primary outcome was time to complete the enterotomy repair in two layers. Secondary outcomes included leak test results, need for additional sutures, and qualitative documentation of technical errors such as suture breakage or incomplete repair.

### Statistical analysis

Continuous variables are presented as mean ± standard deviation. Changes in time to completion across sessions were analyzed using a linear mixed-effects model with participant included as a random effect to account for repeated measures and incomplete follow-up. Session number was treated as a fixed effect.

Paired comparisons between sessions were evaluated using paired t-tests and Wilcoxon signed-rank tests, as appropriate. Binary outcomes, including leak test results, were summarized descriptively. Paired comparisons of leak outcomes across sessions were assessed using exact McNemar testing when applicable.

To evaluate the association between completion time and leak occurrence, a logistic regression model accounting for repeated observations within participants was performed. Leak status (yes/no) was modeled as the binary outcome and completion time as a continuous predictor. Odds ratios (ORs) with 95% confidence intervals (CIs) were calculated.

Exploratory analyses comparing residents with and without prior dedicated robotic laboratory training were performed; these analyses were not powered to detect between-group differences. Statistical significance was defined as a two-sided p value < 0.05. All analyses were conducted using IBM SPSS Statistics (Version 29.0; IBM Corp., Armonk, NY) and GraphPad Prism (Version 10.0; GraphPad Software, San Diego, CA).

### Ethical approval

This study was reviewed and deemed exempt by the Institutional Review Board.

### Informed consent

Informed consent was waived due to the educational nature of the study.

## Results

### Study cohort

A total of 38 PGY-1 and PGY-2 general surgery residents participated in the study. Of these, 26 (68.4%) were PGY-1 residents and 12 (31.6%) were PGY-2 residents, including 16 (42.1%) preliminary residents, and 21 (55.2%) participants were female. Additionally, 22 residents (57.9%) had previously completed a dedicated 1-month robotic training rotation. Participation decreased across sessions, with 5 residents (13.2%) completing one session, 3 (7.9%) completing two sessions, and 30 (78.9%) completing all three sessions.

### Primary outcome: time to completion

**Fig. 1:**
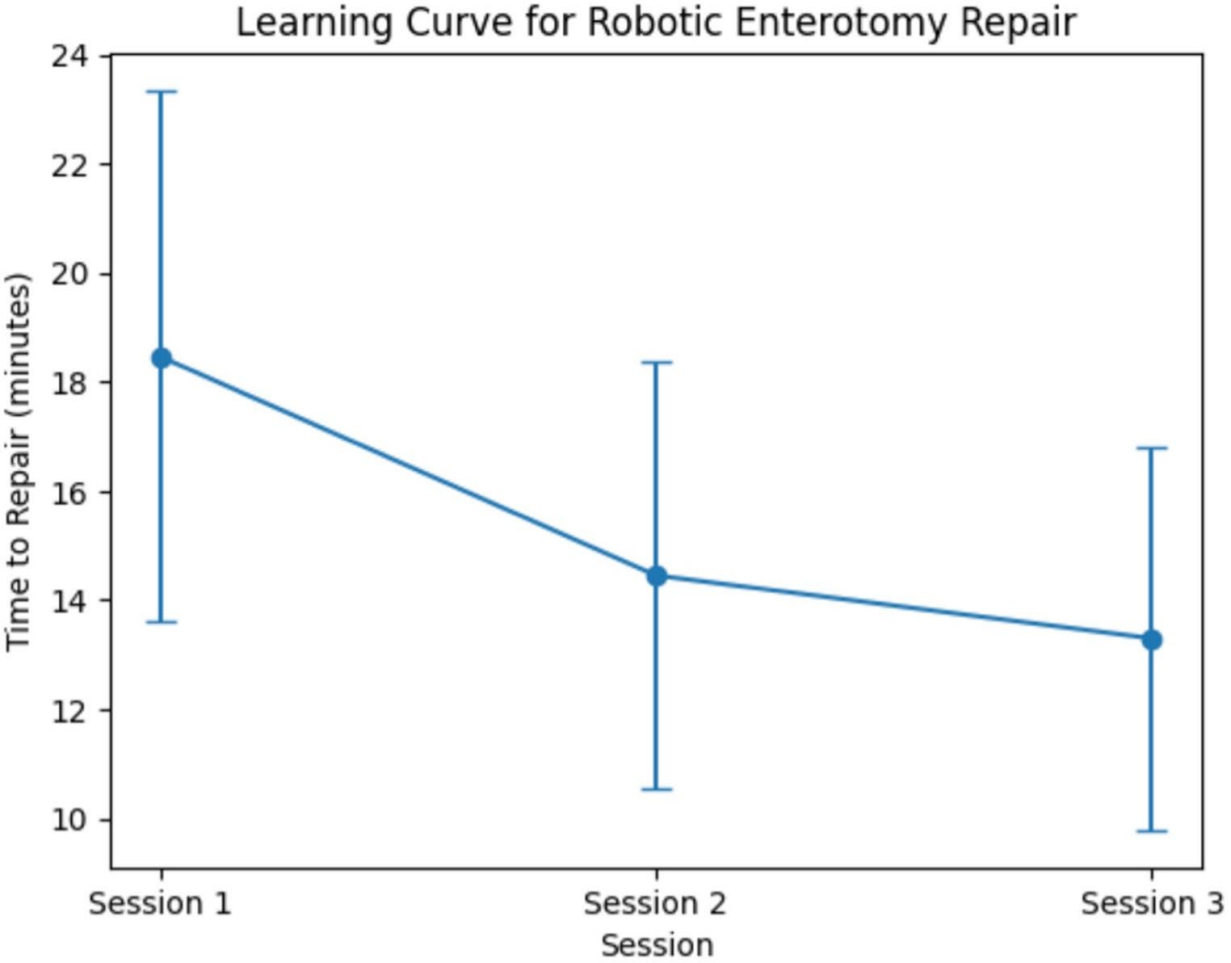
Learning Curve for Robotic Enterotomy Repair [Mean ± SD time to completion across three sessions]. Time to repair decreased significantly across sessions, demonstrating a reproducible learning curve following focused one-on-one instruction

Mean time to completion decreased progressively across sessions (Fig. 1). In Session 1, the mean completion time was 18.47 ± 4.87 min (*n* = 38), which improved to 14.90 ± 6.00 min in Session 2 (*n* = 33) and 13.20 ± 5.60 min in Session 3 (*n* = 30).

Using a linear mixed-effects model with participant included as a random effect, session number was a significant predictor of decreased completion time (*p* < 0.001), demonstrating reproducible improvement in efficiency with repeated participation.

Among participants with paired observations between Sessions 1 and 2, mean completion time improved by 266.35 s (4.44 min; 95% CI 2.64–6.24 min). This improvement was statistically significant by paired t-test (*p* = 3.57 × 10⁻⁵) and Wilcoxon signed-rank test (*p* ≈ 5.32 × 10⁻⁵).

### Reproducibility of skill acquisition

Individual learning curves demonstrated a consistent downward trajectory in both repair time and leak test results, indicating improved performance for the majority of participants. Despite variability in baseline performance, nearly all residents demonstrated measurable improvement by the third session, supporting reproducible skill acquisition rather than isolated performance gains.

### Effect of Prior dedicated robotic laboratory training (exploratory)

Residents with prior dedicated robotic laboratory training demonstrated numerically faster completion times across all sessions compared with residents without prior training. Across sessions, the trained group completed repairs 14.2% faster (effect size: 0.49; 95% CI -0.19–1.17) on average; however, this difference did not reach statistical significance (*p* = 0.16).

Both trained and untrained groups demonstrated improvement over successive sessions. The previously trained group exhibited a more consistent and monotonic learning trajectory, whereas performance among previously untrained residents showed greater variability. In the final session, trained residents completed repairs faster than untrained residents, though this difference was not statistically significant (effect size: 0.39; 95% CI -0.14–0.92, *p* = 0.11).

Leak rates were lower among residents with prior robotic training compared with untrained residents (23.2% vs. 29.6%), representing a 6.4% absolute reduction (95% CI -10.5–23.3%) and a 21.6% relative risk reduction (95% CI -35.1–45.2%); however, this difference did not reach statistical significance (*p* > 0.05). No inferential modeling adjusting for repeated measures was performed for leak outcomes.

### PGY-stratified performance

PGY-1 residents demonstrated greater absolute improvement in time to completion compared with PGY-2 residents. Mean completion time for PGY-1 residents decreased from 19.14 min in Session 1 to 13.26 min in Session 3, representing a mean improvement of 5.88 min (95% CI 3.21–8.55). PGY-2 residents improved from 17.02 min to 13.38 min over the same period, corresponding to a mean improvement of 3.64 min (95% CI 1.02–6.26).

Although PGY-1 residents began with longer completion times, performance converged by Session 3, with comparable final repair times between PGY levels.

### Secondary outcome: leak test performance

**Fig. 2:**
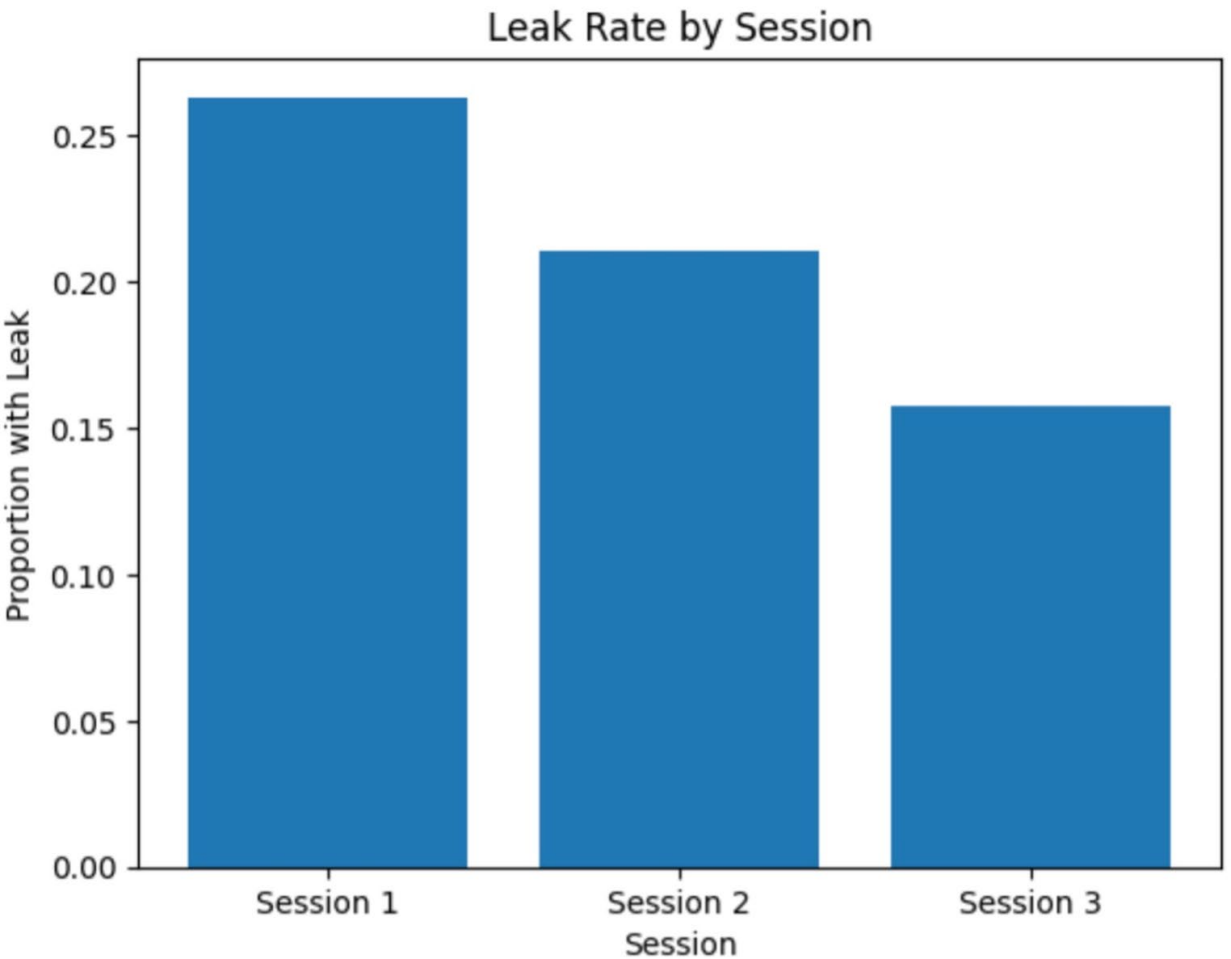
Leak Test Failure Rate by Session [Proportion of repairs failing leak testing across sessions]. Leak rates decreased from 26.3% in session one to less than 20.0% by session three, indicating improved technical integrity with training repetition

Leak test failure rates decreased across successive sessions. In Session 1, 10 of 38 repairs (26.3%) demonstrated a positive leak. Leak rates decreased to 24.2% (8/33) in Session 2 and 20.0% (6/30) in Session 3 (Fig. 2).

**Fig. 3:**
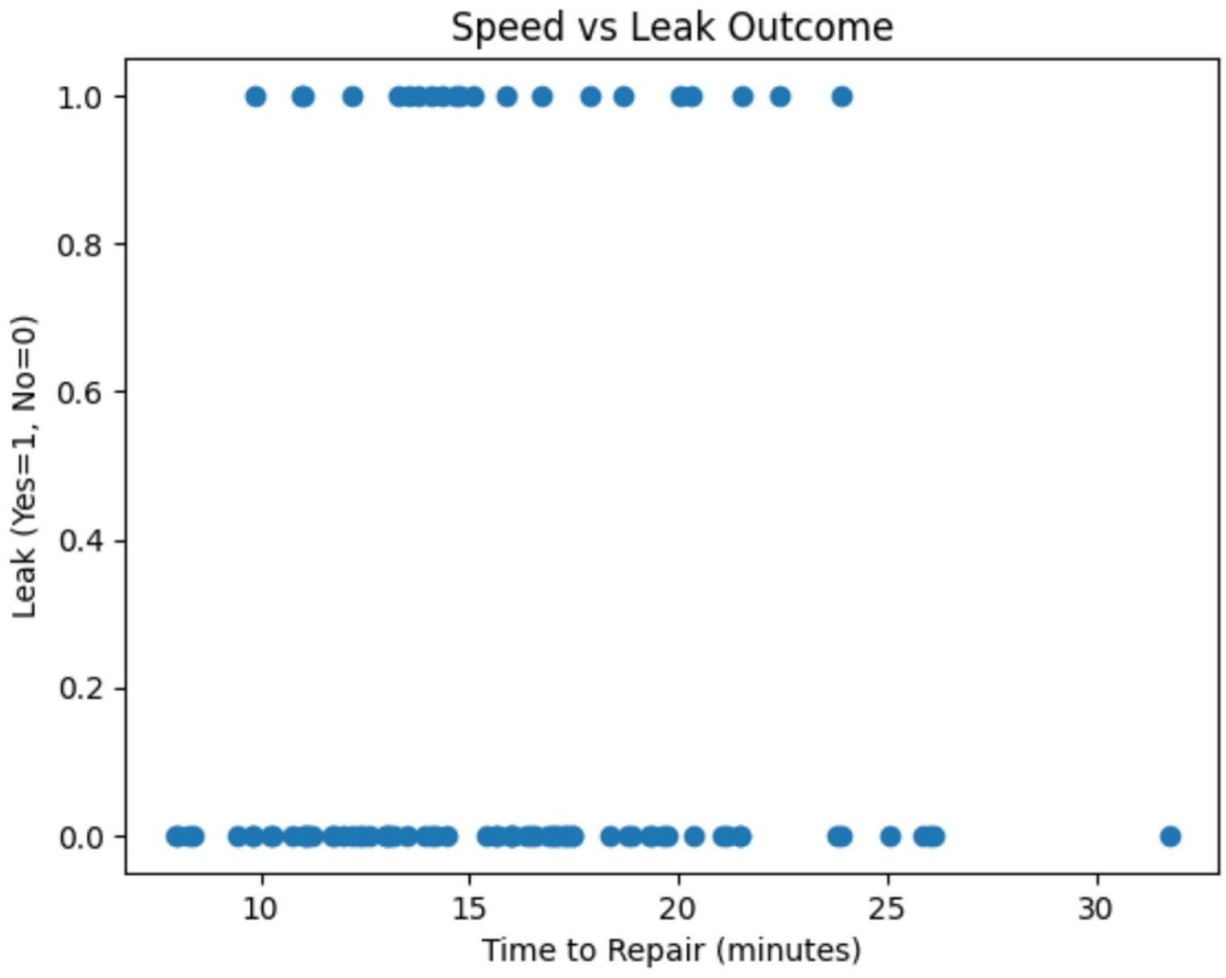
Relationship Between Repair Time and Leak Outcome [Scatter plot of time to completion versus leak outcome across all attempts]. Logistic regression analysis demonstrated no significant association between repair time and leak occurrence (OR 1.0003, 95% CI 0.9988–1.0019, *p* = 0.671), suggesting efficiency gains did not compromise repair quality

Paired comparisons using exact McNemar testing did not demonstrate statistically significant changes in leak rates between sessions. Logistic regression analysis accounting for repeated observations within participants demonstrated no significant association between completion time and leak occurrence (OR 1.0003, 95% CI 0.9988–1.0019, *p* = 0.671). Accordingly, faster completion times were not associated with an increased risk of leak (Fig. 3).

### Technical errors and suture use

Ten participants (26.3%) required more than one barbed or silk suture to complete the repair, and one broken instrument during the task was reported. The frequency of technical errors, including suture breakage and incomplete repairs, decreased across successive sessions, consistent with improving technical proficiency.

## Discussion

In this prospective skills assessment study, we demonstrated that a short, standardized robotic suturing curriculum using a procedure based enterotomy repair task produces rapid, reproducible improvements in technical efficiency among junior surgical residents without compromising repair integrity. Across sessions, residents showed a significant reduction in time to completion accompanied by a downward trend in leak rates, supporting the effectiveness of focused, hands-on robotic training early in residency [13].

These results align with the study by Laverty et al., where senior trainees outperformed juniors in time and error metrics on a robotic enterotomy model, confirming that procedure-based tasks can sensitively detect technical progression [6]. Similarly, Al-Ani et al. observed measurable performance gains across structured simulation modules, reinforcing the value of standardized, metrics driven curricula [10]. Moreover, Kieslich et al.’s recent systematic review and expert consensus highlight simulation, proficiency based modular training, deliberate practice, and objective assessment as fundamental components of an effective robotic surgical curriculum [4].

The importance of deliberate practice, an established concept in psychomotor learning, has been highlighted in multiple publications [14–18], and additional studies emphasize the critical role of feedback in skill acquisition [19–24]. Our study operationalized these principles by combining procedure specific simulation with serial assessment and leak testing, providing a practical example of consensus informed curricular design.

These robotic training studies have largely focused on construct validity using cross sectional comparisons and global rating scales. In contrast, our repeated measures design directly captures learning trajectory and demonstrates skill convergence across training levels. Furthermore, by incorporating leak testing as a functional quality endpoint rather than relying solely on rating scales or error counts, our protocol emphasizes repair integrity in addition to efficiency.

### Reproducible learning with a procedure-based assessment

A central strength of this study is the use of a standardized, clinically relevant task that closely mirrors real intraoperative demands. Unlike abstract simulator metrics or isolated suturing drills, the two-layer robotic enterotomy repair required precise tissue handling, spatial awareness, and knot security, core skills for safe robotic surgery. The consistent reduction in completion time across sessions, confirmed by mixed effects modeling, reflects a reproducible learning curve rather than isolated performance gains. Prior simulator-based curricula often relied heavily on simulation-based analytics [25–27], whereas our procedure-based model emphasizes tissue fidelity and repair mechanics, enhancing translational relevance.

Importantly, improvement was observed despite variability in baseline performance, suggesting that this protocol is capable of detecting meaningful skill acquisition across a heterogeneous trainee population. Nearly all participants demonstrated improvement by the third session, reinforcing the value of repeated, structured exposure even over a relatively short training interval. Comparable heterogeneity was observed in the UK pilot scheme, where junior trainees occasionally outperformed senior residents in specific simulation domains, demonstrating that baseline robotic exposure, not postgraduate year alone, drives performance variability [10].

### Efficiency gains without compromising quality

A frequent concern in surgical training is that increased efficiency may come at the expense of technical quality [13]. In this study, regression analysis accounting for repeated measures demonstrated no significant association between completion time and leak occurrence, suggesting that improvements in efficiency were not achieved at the expense of repair integrity. Leak test failure rates, however, decreased across sessions, indicating progressive improvement in repair integrity with experience. Although paired comparisons of leak outcomes did not reach statistical significance, the observed trend supports the premise that technical refinement and efficiency can improve in parallel. Laverty et al. similarly demonstrated that improved Global Evaluative Assessment of Robotic Skills (GEARS) scores and reduced errors among senior trainees were not achieved at the cost of task fidelity, reinforcing that efficiency metrics can correlate with qualitative performance measures [6].

Leak testing offered an objective, binary measure of repair quality, rarely included in simulation based robotic training. Its inclusion enhances clinical relevance and provides a pragmatic surrogate for real world technical errors. Unlike prior studies using error counts and GEARS scoring, our integration of physiologic leak pressure testing extends previous validation frameworks and moves toward higher fidelity outcome simulation [6].

### Early training and skill convergence

Junior residents, particularly PGY-1 trainees, demonstrated substantial absolute improvements in performance, with convergence of repair times between PGY-1 and PGY-2 residents by the final session, although attrition across sessions occurred due to scheduling conflicts and clinical responsibilities. These findings suggest that early exposure to structured robotic training may mitigate baseline differences in operative experience and accelerate the development of core reconstructive skills. This convergence mirrors observations in other studies, where structured early simulation allowed less senior trainees to perform at levels comparable to more advanced trainees in certain domains [6], which challenge traditional assumptions that robotic proficiency should be deferred until later residency. This has important implications for surgical education, as robotic platforms are increasingly used for complex procedures that demand advanced suturing and tissue handling. Providing early, structured exposure may better prepare trainees for safe participation in robotic cases later in residency.

### Prior robotic training and performance variability

Exploratory analyses suggested that residents with prior dedicated robotic laboratory training completed repairs more efficiently and demonstrated more consistent learning trajectories. Although these differences did not reach statistical significance, likely due to limited power, the observed trends support the educational value of early and repeated robotic exposure. Notably, both trained and untrained residents improved over time, demonstrating the effectiveness of the standardized curriculum itself. These findings are concordant with prior studies, showing that while prior exposure influences baseline efficiency, it does not preclude substantial gains through structured curricula [6, 10].

### Educational implications and generalizability

The robotic bowel suturing protocol described here provides a practical, reproducible framework for assessing robotic skill acquisition. Technically demanding yet feasible, it incorporates both efficiency and quality metrics and can be implemented using readily available simulation resources. This approach may serve as a template for competency-based training, particularly in programs seeking objective benchmarks. Multiple studies have emphasized “benchmarked metrics” as essential for tracking progress and ensuring trainees reach predefined proficiency before advancement [28–35].

The protocol’s simplicity and standardization enhance its generalizability across institutions. By emphasizing a clinically meaningful task rather than abstract simulator metrics, it better aligns training outcomes with operative readiness. This supports broader calls for harmonized, cross-institutional standards, addressing the lack of universally mandated robotic curricula despite rapid platform adoption [6, 10].

### Limitations

This study has several limitations. Participant attrition across sessions may introduce attrition bias; however, mixed-effects modeling was used to account for incomplete follow-up and repeated measures. Participant-specific characteristics, including age, handedness, and prior laparoscopic or robotic experience outside the curriculum, were not systematically collected, and may have contributed to variability in baseline performance and learning trajectories. The study was not designed or powered to detect differences in leak outcomes or to perform subgroup analyses based on baseline experience. While leak testing provides an objective and reproducible measure of repair performance, it does not capture broader dimensions of technical skill, including tissue handling, force application, or long-term anastomotic durability. To minimize evaluator dependence, we focused on objective outcomes (completion time and leak testing) rather than incorporating validated global rating scales such as GEARS; inclusion of such tools in future studies would improve construct validity. Finally, simulation-based performance may not directly translate to operative outcomes, warranting further correlation with intraoperative competency.

### Conclusions

A short, standardized robotic suturing curriculum using a procedure based enterotomy repair task produces rapid, reproducible improvements in technical efficiency among junior surgical residents without compromising repair quality. This protocol offers a practical, objective method for assessing robotic skill acquisition and supports the integration of structured robotic training early in surgical education. By incorporating longitudinal assessment and functional repair integrity metrics, these findings extend prior construct validation studies and provide a scalable framework for early robotic skill development.

## Supplementary Information

Below is the link to the electronic supplementary material.

Supplementary Material 1: Standardized instructional video demonstrating the robotic two-layer enterotomy repair protocol used for participant training prior to testing sessions.

## Data Availability

The datasets generated and/or analyzed during the current study are available from the corresponding author on reasonable request.
